# Possible Correlations between Mean Platelet Volume and Biological, Electrocardiographic, and Echocardiographic Parameters in Patients with Heart Failure

**DOI:** 10.3390/life14020260

**Published:** 2024-02-16

**Authors:** Andreea Catană, Cătălina Liliana Andrei, Suzana Guberna, Octavian Ceban, Crina Julieta Sinescu

**Affiliations:** 1 Department of Cardiology, University of Medicine and Pharmacy “Carol Davila”, Emergency Hospital “Bagdasar-Arseni”, 020021 Bucharest, Romania; sguberna@yahoo.com (S.G.); crina.sinescu@umfcd.ro (C.J.S.); 2 Economic Cybernetics and Informatics Department, The Bucharest University of Economic Studies, 010552 Bucharest, Romania; octavianceban1995@gmail.com

**Keywords:** mean platelet volume, heart failure, NT-proBNP, atrial fibrillation, left ventricular ejection fraction

## Abstract

(1) Background: Despite advancements in medical research and discoveries, heart failure (HF) still represents a significant and prevalent public health challenge. It is characterized by persistently high mortality and morbidity rates, along with increased rates of readmissions, particularly among the elderly population. (2) Methods: This study was conducted retrospectively on 260 patients with stable or decompensated chronic HF. The parameter of interest in the study population was the mean platelet volume (MPV), and the main objective of the research was to identify a possible relationship between MPV and several variables—biological (NT-proBNP, presepsin, red cell distribution width (RDW)), electrocardiographic (atrial fibrillation (AFib) rhythm, sinus rhythm (SR)), and echocardiographic (left ventricle ejection fraction (LVEF), left atrial (LA) diameter, left ventricle (LV) diameter, pulmonary hypertension (PH)). (3) Results: By applying logistic and linear regression models, we assessed whether there is a correlation between MPV and biological, electrocardiographic, and echocardiographic variables in patients with HF. The results revealed linear relationships between MPV and NT pro-BNP values and between MPV and RDW values, and an increased probability for the patients to have an AFib rhythm, reduced LVEF, dilated LA, dilated LV, and PH as their MPV value increases. The results were deemed statistically relevant based on a *p*-value below 0.05. (4) Conclusions: Through regression model analyses, our research revealed that certain negative variables in HF patients such as increased levels of NT-proBNP, increased levels of RDW, AFib rhythm, reduced LVEF, dilated LA, dilated LV, and PH, could be predicted based on MPV values.

## 1. Introduction

Cardiovascular diseases still represent a leading cause of mortality globally. The incidence of diagnosed cases is on the rise across all countries, irrespective of their socioeconomic status. Furthermore, the global prevalence of cardiovascular diseases doubled in 2019 in comparison with 1990 [1,2].

Despite the increasingly efficient methods of investigations and the most complex treatment schemes worldwide through treatment guidelines, the number of deaths from cardiovascular causes has increased by approximately 66% [3].

The increase in life expectancy worldwide is one of the causes of the situations presented above. This has caused the burden of cardiovascular disease for each individual to increase [3].

Within the large spectrum of cardiovascular diseases, heart failure (HF) stands out as a significant and prevalent public health concern, with high rates of mortality, morbidity, and hospitalization. It maintains a leading position among cardiovascular conditions with a substantial negative impact on quality of life. This is evident through diminished exercise capacity and constraints on individual physical exertion, marking it as one of the cardiovascular diseases associated with the greatest degree of disability [4].

The role of platelets in hemostasis is very well known, but they also have an important role in the pathogenesis of atheroma plaque and atherothrombosis, and among platelet indices, mean platelet volume (MPV) is a biomarker through which platelet reactivity can be quantified [5,6,7].

The size of a platelet is directly correlated with its level of activity, hence larger platelets are inherently more metabolically active and contain a greater quantity of prothrombotic material [5,6,7]. Research studies have demonstrated a noteworthy relationship between MPV and cardiovascular risk factors, as well as an association with the risk of cardiovascular disease and cardiovascular-related mortality [5,6,7].

Platelet irregularities and dysfunction observed in individuals with heart failure are a consequence of elevated P-selectin expression, platelet aggregation, the presence of platelet-derived adhesion molecules, and increased mean platelet volume [8,9].

An elevated mean platelet volume in individuals with heart failure could be explained by an increase in platelet activity because of the activation of compensatory mechanisms launched in heart failure, which are represented by increased catecholaminergic secretion, increased activity of the renin–angiotensin system, and increased activity of the inflammatory system [10,11]. The link between MPV elevation and the activation of compensatory mechanisms in heart failure is outlined in a study conducted by Karabacak et al., who investigated the effects of beta-blockers on MPV and on the prognosis of heart failure. The results of the aforementioned study revealed that patients with heart failure who received beta-blockers experienced a decrease in their MPV levels and a better outcome, the possible explanation for this phenomenon being that beta-blockers decrease catecholaminergic activity and thus decrease the levels of MPV [12]. 

And since these compensatory mechanisms are launched in heart failure patients at risk of decompensation, this explains why the mean platelet volume (MPV) appears increased in those individuals. This hypothesis was tested in a study conducted by Hakki Kaya on patients with heart failure, which revealed that MPV was increased in the group of patients who were rehospitalized for decompensation [3].

Another possible hypothesis for why mean platelet volume increases in heart failure could be explained by the fact that heart failure patients are frequently predisposed to paroxysmal episodes of atrial fibrillation, an arrhythmia that is correlated with increased levels of MPV according to the study conducted by Turgut O et al. [13].

Studies on the relationship between the MPV value and certain biological (presepsin, NT-proBNP, red cell distribution width (RDW)), echocardiographic (left ventricle ejection fraction (LVEF), left atrium (LA) dimensions, left ventricle (LV) dimensions, presence of pulmonary hypertension (PH)), and electrocardiographic (sinus rhythm (SR), atrial fibrillation (AFib) rhythm) parameters in groups of subjects with HF are limited.

Using the MPV value as a predictor for certain negative variables in patients with HF such as reduced LVEF, increased NT-proBNP value, dilated LA, dilated LV, the presence of PH, and the presence of AFib rhythms is an element of novelty that could assist practitioners in identifying HF patients predisposed to a negative prognosis. Consequently, it may enable the implementation of more effective and potentially aggressive therapeutical plans for these individuals. 

Based on the limited available medical literature concerning this issue, through our research, we aim to identify possible correlations between MPV and certain biological, electrocardiographic, and echocardiographic variables in patients with HF. Based on the above correlations that we aim to find, MPV could be used as a prognostic parameter in populations of patients with HF, and through this, we could gain the possibility to identify patients with HF at risk of having a negative prognosis. This finding could represent a new element of interest brought to the medical literature regarding new prognostic parameters that could be used in HF patients.

## 2. Methods

### 2.1. Design of the Study and Patient Selection

Our study was conducted retrospectively on patients with stable or decompensated chronic heart failure (HF) who were admitted to the Cardiology Department of the “Bagdasar-Arseni” Emergency Clinical Hospital. The main variable of interest in this study population was the mean platelet volume (MPV), and the main objective of our research was to identify a possible relationship between MPV and certain variables of interest that will be described in the paragraphs below.

According to the 2021 ESC Guidelines for HF [14], decompensated chronic HF is defined as the worsening of patients’ clinical status, being included in NYHA functional classes III/IV, which are defined by a sum of symptoms and signs represented by symptoms at rest and signs of congestion, whereas patients with stable chronic HF are defined as individuals with a personal history of heart failure, who are asymptomatic or who have slight limitations of physical activity (NYHA functional classes I/II).

Regarding the sample size estimation, we included in our database all patients with heart failure admitted to the Cardiology Department of the Emergency Clinical Hospital Bagdasar-Arseni between January 2017 and January 2019, of which we selected 260 patients who met the inclusion criteria, which were as follows: patients with chronic HF (stable or decompensated), patients whose MPV value was available, and patients for which data on biological (RDW, NT-proBNP, presepsin), electrocardiographic (AFib, SR), and echocardiographic parameters (LA diameter, LV diameter, LVEF, PH) were available. 

The patients with heart failure admitted in that period who were not included in the study met the exclusion criteria, which were as follows: patients without heart failure, subjects on which biological, electrocardiographic, and echocardiographic data for the variables of interest were not available, and patients who had diseases with a low survival time, active cancer, acute or chronic kidney disease, acute coronary syndrome, stroke, acute pulmonary embolism, or abnormal platelets levels/hematological diseases.

### 2.2. Data Collection

Our prime objective was to explore whether MPV could serve as a potential predictor factor in a study population consisting of patients with HF. Using logistic and linear regression models, our statistical analyses aimed to determine if there is a relevant relationship between the MPV value and specific categories of variables including biological factors (NT-proBNP, presepsin, RDW), electrocardiographic measures (AFib rhythm, SR), and echocardiographic measures (LVEF, LA diameter, LV diameter, PH). 

Clinical and paraclinical data of patients with HF were obtained through medical records and by using the System Database of the “Bagdasar-Arseni” Emergency Clinical Hospital when the needed data were not available in the medical records.

Data regarding biological (NT-proBNP, presepsin, RDW), electrocardiographic (heart rhythm), and echocardiographic markers (left and right cavities’ dimensions, left ventricular ejection fraction (LVEF), presence or absence of pulmonary hypertension) were obtained from the hospital’s medical records.

The values of NT-proBNP considered significant for cardiac decompensation according to the ESC 2021 Guideline of HF [14] are as follows: over 450 pg/mL in patients under the age of 55, over 900 pg/mL in patients over the age of 55 but under 75 years, and over 1800 pg/mL in patients over 75 years old. A presepsin value that is considered significant for a possible infection is any value above 300 mg/dl. The RDW value is considered high over a value of 14%.

Regarding the cavity dimensions, according to the EACVI (European Association of Cardiovascular Imaging) guidelines [15], a left ventricle (LV) in men is considered dilated at an LV end-diastolic diameter (LVTDD) of more than 50 mm, and in women, at an LVTDD of more than 40 mm; the left atrium (LA) is considered dilated if the indexed LA volume is over 34 mL/sc, regardless of the patient’s sex. According to EACVI guidelines [16], the LVEF is considered preserved at over 51%, slightly reduced at 41–50%, moderately reduced at 30–40%, and severely reduced when below 30%. Regarding pulmonary hypertension (PH), according to the ESC 2022 Guidelines on PH [17], a patient is considered to have an intermediate probability of PH in the presence of a peak tricuspid velocity (PTV) of 2.9–3.4 m/s or a PTV under 2.9 m/s plus other echo PH signs, and a patient is considered to have a high probability of PH in the presence of a PTV of 2.9–3.4 m/s plus other PH echo signs, or in the presence of a PTV over 3.4 m/s.

The basal values of some of these variables are presented in Table 1.

### 2.3. Statistical Analyses

The database was conceived using the Excel program, and the statistical analyses were performed based on logistic and linear regression models, which were processed in Python 3.10.

We used linear regression for cases where the dependent variable was continuous: NT-proBNP, presepsin, RDW, LVEF, LA diameter, LV diameter; and logistic regression where it was a classification problem (binary outcome): AFib, SR, reduced LVEF, LVEF preserved, dilated LA, dilated LV, PH. 

In the case of the linear regression, we estimate a model in the form of $y=\beta_{0}+\beta_{1}*x_{1}+\varepsilon$, where *y* is the dependent continuous variable and $x_{1}$ is the MPV (independent variable). $\beta_{0}$ and $\beta_{1}$ are the parameters that are going to be estimated on the sample data. By fitting a model like this, we can come up with interpretations about the relationship between *x* and *y*: for example, by testing the significance of $\beta_{1}$, which tells us whether we have reason to believe that there is a true relationship in the population level between *x* and *y*.

For the logistic regression, the obtained model is similar, and is defined as $score=\beta_{0}+\beta_{1}*x_{1}+\varepsilon$, where *score* is the probability that the event will happen (*y* = dependent binary variable), $x_{1}$ is the MPV value (independent variable), and $\beta_{0}$ and $\beta_{1}$ are the parameters that are going to be estimated on the sample data ($\beta_{0}$ = the coefficient for the dependent variable, $\beta_{1}$= the coefficient for the MPV).

The logistic interpretations are very similar to those for the linear regression, except for the fact that, since we are predicting the outcome of an event, it is very useful to compute the estimated probability of that event to happen. Therefore, the probability of an event happening *y* is estimated based on the following equation: $probability=\frac{1}{1+e^{-score}}$. 

The model was deemed statistically relevant at a *p*-value less than 0.05.

We have attached an Appendix A below that reveals all of the variables used in our research.

## 3. Results

Among the patients with heart failure admitted between 2017 and 2019, we included 260 patients who met the inclusion criteria mentioned above. 

Regarding the patients’ characteristics, of the 260 patients with heart failure, 71% had NYHA class IV heart failure, 15% had NYHA class III, and 14% had NYHA class I/II; 58% were female patients and 42% were male patients; 3% were aged between >40 and <50 years, 12% were aged between ≥50 and <60 years, 24% were aged between ≥60 and <70 years, and 61% were aged ≥70 years.

Concerning the correlation between the MPV value and biological (NT-proBNP, presepsin, RDW), electrocardiographic (AFib rhythm, SR), and echocardiographic parameters (LVEF, LA diameter, LV diameter, PH), detailed statistical data are presented in the subsequent paragraphs. 

### 3.1. Biological Variables (NT-proBNP, Presepsin, RDW)

#### 3.1.1. MPV–NT-proBNP Relationship

Concerning the relationship between MPV and the NT-proBNP value in our study population, our findings indicated that an elevated MPV is associated with a higher NT- proBNP value. This association was established using the linear regression model and the data regarding the MPV coefficients are revealed in Table 2. 

The data presented in Table 2 reveal the following key insights:

(1) The value of 0.091 associated with the R-square signifies that the changes in the value of NT-proBNP by 9.1% are explained by the changes in the value of MPV.
(2) The value of 2677.3154 associated with the MPV coefficient indicates that when the MPV increases by one unit, the NT-proBNP value increases by 2677 pg/mL.
(3) The *p*-value = 0.000, thus the model is statistically relevant, signifying that a higher MPV value is indeed linked to a higher NT-proBNP value.

**Figure 1:**
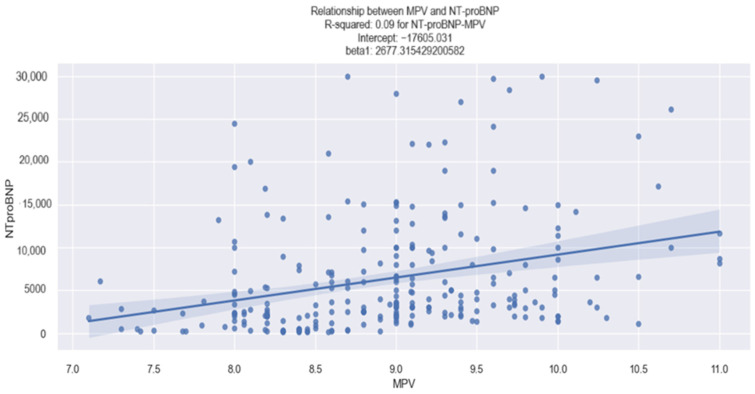
Linear relationship between MPV and NT-proBNP value in HF patients.

This hypothesis, according to which there is a linear relationship between MPV and NT-proBNP, is depicted in Figure 1. The graph shows a tendency for the NT-proBNP value to rise as the MPV value increases. As an example, in a patient with an MPV value of 10.5 fl, an associated NT-proBNP value over 10.000 pg/mL can be anticipated. 

#### 3.1.2. MPV–Presepsin Relationship

Concerning the relationship between MPV and the presepsin value in patients with HF, by using the linear regression model, whose estimated coefficients are revealed in Table 3, our results show that there was not a relevant correlation between MPV and presepsin values. In this case, the *p*-value is at the limit of the threshold value of a statistically significant *p*-value (0.05), therefore we cannot assert with sufficient certainty that a higher MPV value is associated with an elevated presepsin value. These results can also be explained by the fact that the presepsin blood sample was not available for all of our patients, with the number of patients for whom presepsin data were available being 39.

**Figure 2:**
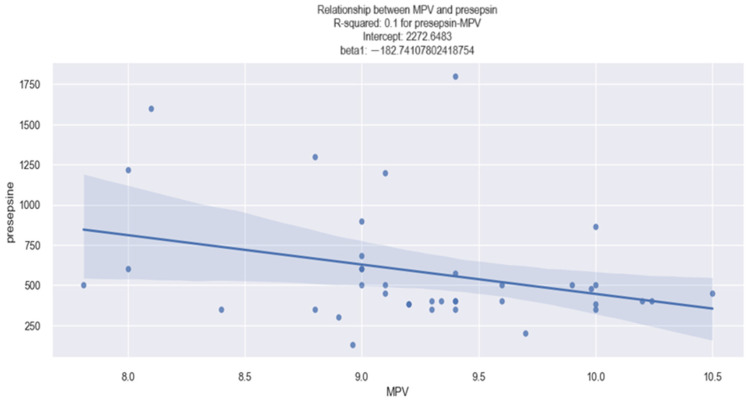
Linear relationship between MPV and presepsin value in HF patients.

Due to the fact that in our sample of patients with HF there was not a very large number of patients with available presepsin data, it was not possible to identify the existence of a relevant linear relationship between the MPV value and presepsin value. The depicted figure (Figure 2) illustrates somewhat contradictory findings. Contrary to our expectations, patients with higher MPV values exhibited a tendency towards lower presepsin values, rather than the anticipated trend of higher presepsin values. This observation contradicts the statistical results commonly reported in the medical literature regarding the correlation between MPV and sepsis. 

#### 3.1.3. RDW–MPV Relationship

Concerning the relationship between the MPV and red cell distribution width (RDW) values in our study, our findings indicate that an elevated MPV is associated with a higher RDW value. This association was established through the application of the linear regression method, and the data regarding the MPV coefficients are revealed in Table 4. 

The data presented in Table 4 reveal the following key insights:

(1) The value of 0.037 associated with the R-square signifies that the change in the value of RDW by 3.7% are explained by the change in the value of MPV.
(2) The value of 0.43 associated with the MPV coefficient indicates the following: an increase of one unit in the MPV value is associated with an average increase of 0.4377% in the RDW value.
(3) The *p*-value associated with the MPV coefficient (0.43) is 0.002, which equals a high level of confidence in stating that patients with a higher MPV value upon admission had a higher RDW value.

**Figure 3:**
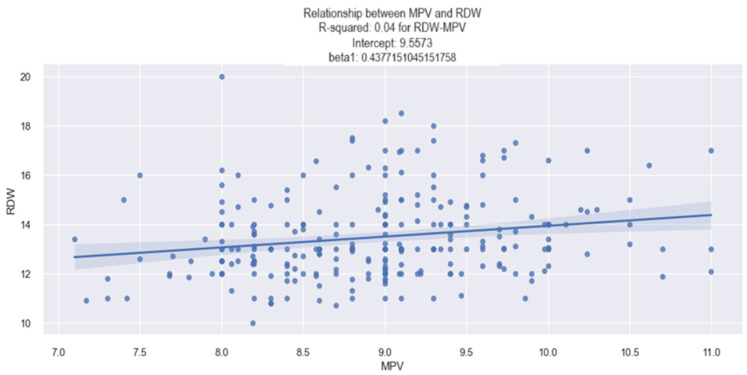
Linear relationship between MPV and RDW value in HF patients.

The linear relationship between the MPV value and the RDW value is visually depicted in Figure 3. The graph illustrates a slight tendency for the RDW to increase as the MPV value rises. For instance, in a patient with an MPV value of 10 fl, an associated RDW value of 14% can be expected. 

### 3.2. Electrocardiographic Parameters—Atrial Fibrillation and Sinus Rhythm

#### 3.2.1. MPV–Atrial Fibrillation Relationship

**Figure 4:**
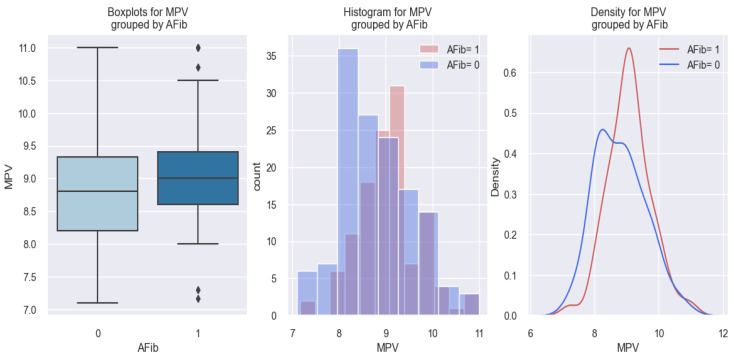
Differences in MPV values between subjects with AFib (1) and without AFib (0).

Concerning the relationship between MPV and atrial fibrillation (AFib), statistical results showed that patients discovered at admission to have an AFib rhythm had a higher MPV value compared to those without AFib. These data are graphically represented in Figure 4. In the first graph of Figure 4, it can be seen that patients with an AFib rhythm (AFib 1) had a higher MVP value (9 fl) compared to those without AFib (AFib 0), who had MPV values below 9 fl. Similar results are represented in the following two graphs, where it is observed that there is a tendency for patients with AFib (AFib 1) to be distributed more to the right, thus more towards higher values of MPV.

The findings from the above results are effectively explained through the application of the logistic regression model. The estimated coefficients of this model are presented in Table 5. This model allowed us to estimate the probability of a patient with HF having an AFib rhythm based on their MPV value. 

The applied model, in this case, is represented as follows: score (event occurrence = AFib) = −4.8282 + 0.5261*MPV. Using this formula, if we consider having a patient with an MPV of 7 fl, we obtain a score of –1.14, and if we consider having a patient with an MPV of 10 fl, we obtain a score of 0.43. By applying the following equation: $probability=\frac{1}{1+e^{-score}}$, we can convert these results into probabilities. Therefore, in a patient with an MPV of 7 fl, the chances of having AFib are low (0.24 or 24%), and in a patient with an MPV of 10 fl, the chances of having AFib are high (0.60 or 60%). The *p*-value associated with this model is 0.003; thus, the mathematical model described above is considered relevant. 

Therefore, based on the arguments presented, we may assert that the MPV value allows us to estimate the likelihood of a patient with HF having an AFib rhythm and that the higher their MPV value, the higher the likelihood of patients with HF having AFib rhythms. While the pseudo-R-squared is 2.6%, indicating that a relatively small proportion of AFib events can be explained by the MPV value, there is still a statistically significant correlation between MPV values and AFib rhythms. 

**Figure 5:**
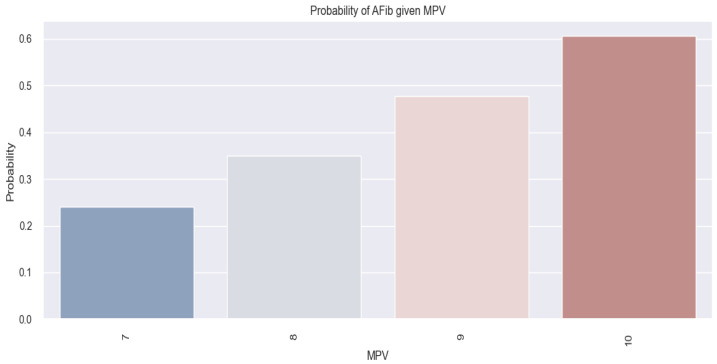
Estimated probability of having AFib depending on MPV value in the studied population.

The conclusions derived from the results above are also depicted in Figure 5, illustrating an increasing probability trend of AFib presence as the MPV value rises.

#### 3.2.2. MPV–Sinus Rhythm Relationship

**Figure 6:**
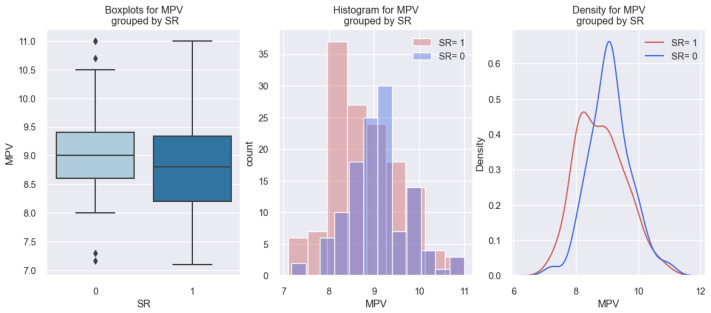
Differences in MPV values between subjects with SR (1) and without SR (0).

In our investigation of the relationship between MPV and sinus rhythm (SR) in the studied population, our study results exhibited an opposite trend in comparison to patients with AFib rhythms. The data from our research revealed that patients with SR on admission exhibited lower MPV values in comparison to those without SR: these results are practically “mirror-like” compared to the patients with AFib on admission. The data obtained regarding this phenomenon are represented graphically in Figure 6. From the first graph of Figure 6, it is observed that patients with SR (SR 1) have a lower value of MPV compared to those without SR (SR 0)—8.8 versus 9 fl—and in the following two graphs, patients with SR (SR 1) are distributed more to the left, i.e., towards lower values of MPV (below 9 fl).

The above results are outlined through the application of the logistic regression model, and Table 6 shows the data regarding the model coefficients. This model allowed us to determine the likelihood of a patient in the studied population having SR depending on their value of MPV. 

**Figure 7:**
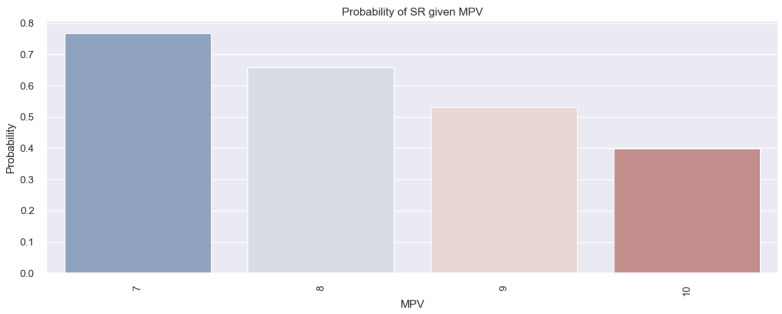
Estimated probability of having SR depending on MPV value in the studied population.

The model in this case is represented as follows: (event occurrence = SR) = 4.9591 − 0.5372*MPV. Using this formula, if we consider having an MPV of 7 fl, we obtain a score of −0.40. By applying the following equation: $probability=\frac{1}{1+e^{-score}}$, we can convert these results into probabilities. Therefore, for a patient with an MPV of 7 fl, the chances of them having SR are high (0.78 or 78%). The *p*-value associated with this model is 0.002; therefore, the mathematical model described above is considered relevant. The data derived from this model are also illustrated in Figure 7, which reveals that a decreased MPV in the study population is associated with a high chance of having SR. In summary, based on the results above, we deduced that MPV allows us to estimate the likelihood of having SR in our studied population and that the lower the MPV value, the greater the likelihood of HF patients having SR. 

### 3.3. Echocardiographic Parameters (Left Ventricular Ejection Fraction, Left Atrium, Left Ventricle, Pulmonary Hypertension)

#### 3.3.1. MPV–Left Ventricular Ejection Fraction Relationship

Regarding the relationship between the MPV value and the left ventricular ejection fraction (LVEF), the statistical data revealed promising results. The linear relationship between MPV and the LVEF value in patients with HF in our study was identified by applying the linear regression model (Table 7), through which we found that as the MPV value increases, the LVEF value decreases. This finding was derived from an analysis of the data presented in the table below (Table 7), revealing the following:

(1) The value of 0.053 associated with the R-square signifies that the changes in the value of LVEF by 5.3% are explained by the changes in the value of MPV.
(2) The value of −3.9016 associated with the MPV coefficient indicates that when the MPV increases by one unit, the LVEF value decreases by 3.9016%.
(3) The *p*-value = 0.000, thus the model is statistically relevant.

Therefore, based on the linear regression model, it can be concluded that there is a relevant relationship between the MPV value and LVEF value in patients with HF. 

**Figure 8:**
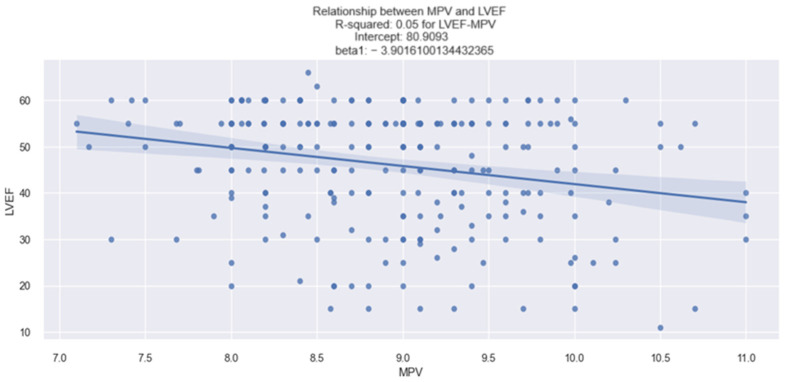
Linear relationship between MPV and LVEF in patients with HF.

The conclusion obtained from the analysis of the above data is depicted in Figure 8, which is a graphical representation of the linear relationship between the MPV and LVEF values, and from which we can observe the tendency of the LVEF value to decrease as the MPV value increases (for example, an MPV value of 11 fl corresponds to an LVEF value, on average, below 40%).

##### MPV–Reduced LVEF Relationship

According to the ESC 2021 HF Guidelines [14], HF cases are classified according to the LVEF value as HF with a preserved LVEF (LVEF > 50%), HF with a medium-reduced LVEF (LVEF > 40–<50%), or HF with a severely reduced LVEF (LVEF < 40%).

In addition to the statistically significant relationship between the MPV and LVEF values, the statistical data of our study revealed varied and significant results regarding the type of LVEF (reduced versus preserved) and MPV value, the results being presented in the paragraphs below.

**Figure 9:**
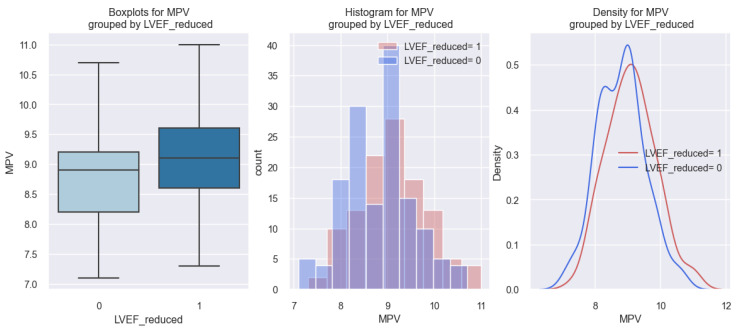
Reported MPV values in HF patients with a reduced LVEF (LVEF reduced = 1) versus HF patients without a reduced LVEF (LVEF reduced = 0).

Regarding the relationship between MPV and the presence of a reduced LVEF (r LVEF) in patients with HF, it was found that patients with r LVEF had a higher value of MPV compared to those without r LVEF; these data are represented graphically in Figure 9.

In the first graph of Figure 9, it can be seen that patients with r LVEF (1) had a higher MPV value (approximately 9.2 fl) in comparison to those without r LVEF (0), who had MPV values below 9 fl. From the following two graphs, a tendency can be observed for the patients with r LVEF (1) to be distributed more to the right, thus towards higher values of MPV.

The above results are outlined through the application of the logistic regression model, and Table 8 shows the data regarding the model coefficients. This model allowed us to determine the possibility of a patient having a reduced LVEF in our studied population depending on their value of MPV. 

The model, in this case, is represented as follows: (event occurrence = reduced LVEF) = −5.1414 + 0.5485*MPV. Using this formula, if we consider having an MPV of 10 fl, we obtain a score of 0.34. By applying the following equation: $probability=\frac{1}{1+e^{-score}}$, we can convert these results into probabilities. Hence, for a patient with an MPV of 10 fl, the chances of them having a reduced LVEF are high (0.59 or 59%). 

**Figure 10:**
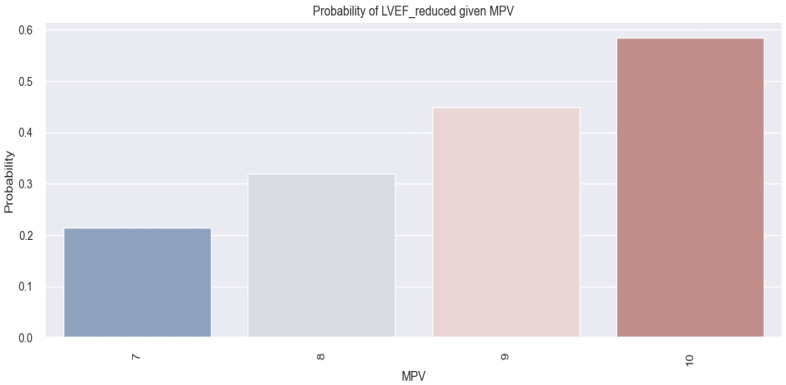
Estimated probability of having reduced LVEF depending on MPV value in the studied population.

The above mathematical model is statistically relevant (*p*-value = 0.002). The data derived from this model are also illustrated in Figure 10, where it is observed that an increased MPV in this study population is associated with a high chance of having a reduced LVEF. In summary, based on the results above, we deduced that the MPV allows us to estimate the probability of having a reduced LVEF in our studied population and that the higher the MPV value, the greater the likelihood of an HF patient having a reduced LVEF. 

##### MPV–Preserved LVEF Relationship

**Figure 11:**
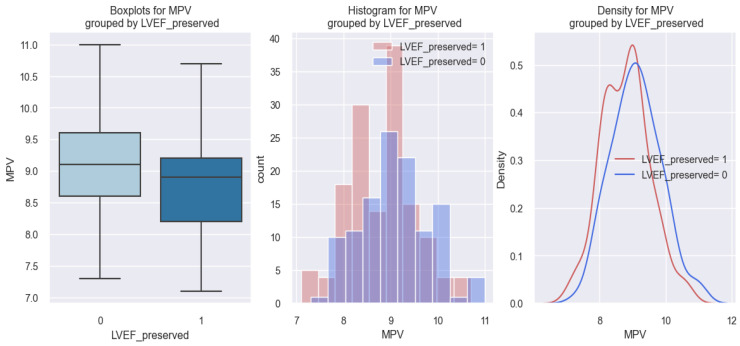
Reported MPV values in patients with HF with a preserved LVEF (LVEF preserved = 1) versus patients with HF without a preserved LVEF (LVEF preserved = 0).

Regarding the statistical data related to the relationship between MPV and preserved LVEF (p LVEF), the results are opposite to those obtained in the case of patients with a reduced LVEF. The results of our study showed that patients with p LVEF had a lower value of MPV compared to those without p LVEF, as can be seen in the graphs represented in Figure 11. From the first graph of Figure 11, it can be seen that patients with p LVEF (1) had a lower MPV value (8.8 fl) compared to those without p LVEF (0), who had MPV values above 9 fl, and on the following two graphs, the distribution tendency of the patients with p LVEF (1) can be observed as veering towards the left, so towards lower MPV values.

The graphical representation of the above findings was delineated through the application of the logistic regression model. The estimated coefficients for this model are presented in Table 9. This model allows us to assess the chance of an HF patient having a preserved LVEF, depending on their MPV.

The applied model in this instance is expressed as follows: score (event occurrence = preserved LVEF) = 5.4001 − 0.581*MPV. Given this model, we can consider that, for example, a patient with an MPV of 8 fl has a score of 0.7521. In order to convert these scores into probabilities, we use the following equation: $probability=\frac{1}{1+e^{-score}}$. Consequently, the probability of a patient with an MPV of 8 fl having p LVEF is calculated to be 0.69, or 69%. The mathematical model applied to HF patients is relevant in this case (*p*-value = 0.001).

**Figure 12:**
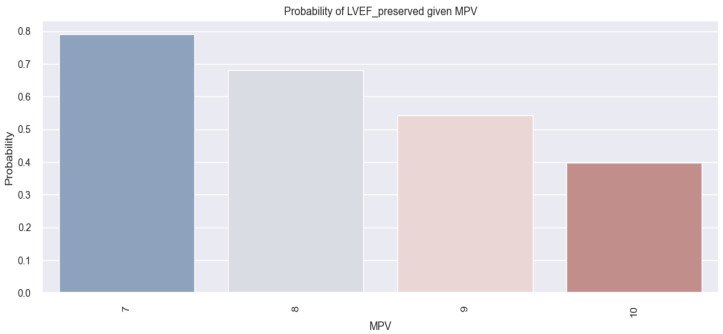
Estimated probability of having a preserved LVEF depending on MPV value in the studied population.

Figure 12 illustrates the previously derived results, indicating a trend wherein HF patients exhibit an increased probability of having a preserved LVEF as their MPV value decreases.

Corroborating the previously mentioned statistical data, we may assert that based on the value of a patient’s MPV, we can predict the possibility of them having a preserved LVEF: the lower the value of MPV, the higher the probability of a patient with HF having a preserved LVEF.

#### 3.3.2. MPV–Left Atrium Size Relationship

The linear relationship between the MPV value and the dimensions of the left atrium (LA), expressed in mm, in patients with HF in our study was identified by applying the linear regression method (Table 10), which showed that as the MPV value increases, the dimensions of the LA also increase. This finding was obtained from our analysis of the data presented in Table 10, which shows the following:

(1) The coefficient for MPV is 2.3035, indicating that, on average, when the MPV increases by one unit, the LA diameter increases, on average, by 2.3 mm.
(2) The associated *p-value* for the MPV coefficient is 0.000, signifying a statistically relevant result. Therefore, it can be affirmed that there exists a noteworthy relationship between the MPV value and the LA diameter in HF patients.

**Figure 13:**
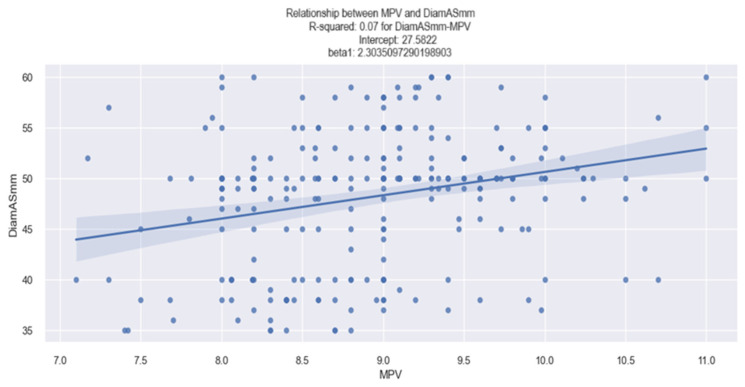
Linear relationship between MPV and LA diameter in patients with HF.

The conclusion obtained from the analysis of the above data is outlined in Figure 13, which depicts a graphical representation of the linear relationship between the MPV value and the LA diameter, and which shows that there is a tendency of the LA diameter to increase as the MPV value increases.

**Figure 14:**
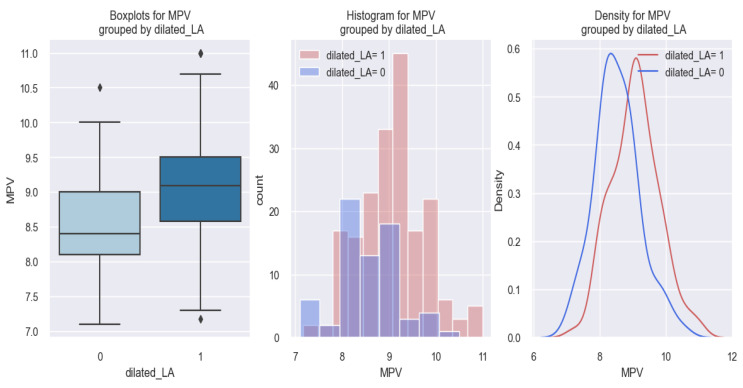
MPV values reported in HF patients with a dilated LA (dilated LA = 1) versus HF patients without a dilated LA (dilated LA = 0).

**Figure 15:**
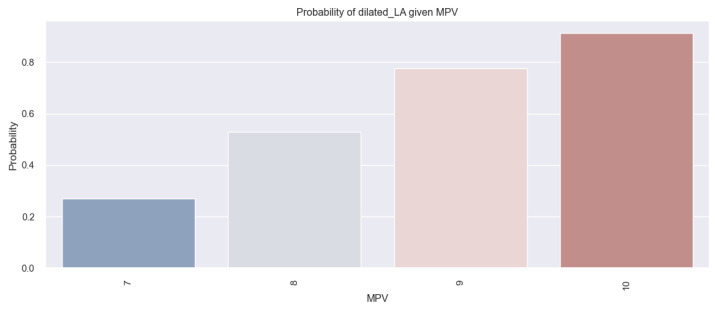
Estimated probability of HF patients having a dilated LA based on their MPV value, using the logistic regression method.

At the same time, the relationship between a higher MPV value and a dilated LA is exemplified clearly in the figures below (Figure 14 and Figure 15).

In the first graph of Figure 14, it can be seen that patients with a dilated LA (1) had a higher MPV value (about 9.1 fl) in comparison to those with a non-dilated LA (0), who had MPV values below 9 fl (8.4 fl), and in the following two graphs, a tendency was observed for patients with a dilated LA (1) to be distributed more to the right, thus towards higher values of MPV.

The aforementioned results are more effectively illustrated through the application of the logistic regression model. The graphical representation of this model is depicted in Figure 15, which reveals that for HF patients with an increased MPV, there is a tendency of an increased probability of them having a dilated LA (for example, there is an over 80% chance for a patient with an MPV of 10 fl to have a dilated LA, versus a patient with an MPV of 8 fl, for whom this chance is under 60%).

In summary, considering the arguments presented, it can be affirmed that the MPV value serves as a predictor for the probability of an HF patient to have a dilated LA. Moreover, our results reveal that the higher the value of their MPV, the greater the probability that a patient with HF will have a dilated LA.

#### 3.3.3. MPV–LV Dimension Relationship

The linear relationship between the MPV and the dimensions of the left ventricle (LV), expressed in mm, in patients with HF in our study was identified by applying the linear regression method (Table 11), which showed that as the MPV value increases, the LV dimensions also increase. This finding was obtained from an analysis of the data in the table below (Table 11), which shows the following:

(1) The coefficient for the MPV is 1.6395, meaning that when the MPV increases by one unit, the LV diameter increases, on average, by 1.63 mm.
(2) The associated *p-value* for the MPV coefficient is 0.000, signifying a statistically relevant result. Thus, it can be affirmed that there exists a significant relationship between the MPV value and the LV diameter in HF patients from our study.

**Figure 16:**
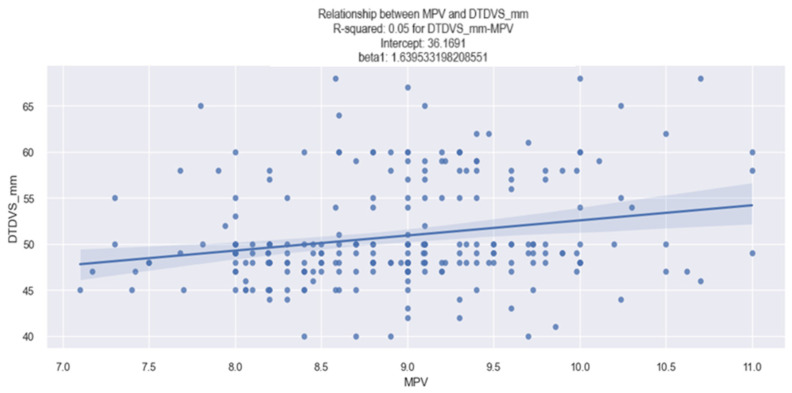
Linear relationship between MPV and LV diameter in patients with HF.

The conclusions obtained from the analysis of the data above are depicted in Figure 16, which is a graphical representation of the linear relationship between the MPV value and the LV diameter, and from which we can observe a tendency of the LV diameter to increase as the MPV value increases.

**Figure 17:**
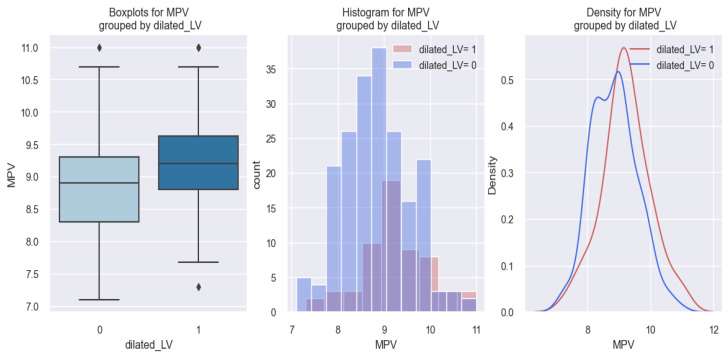
MPV values reported in patients with a dilated LV (dilated LV = 1) versus HF patients with a non-dilated LV (dilated LV = 0).

**Figure 18:**
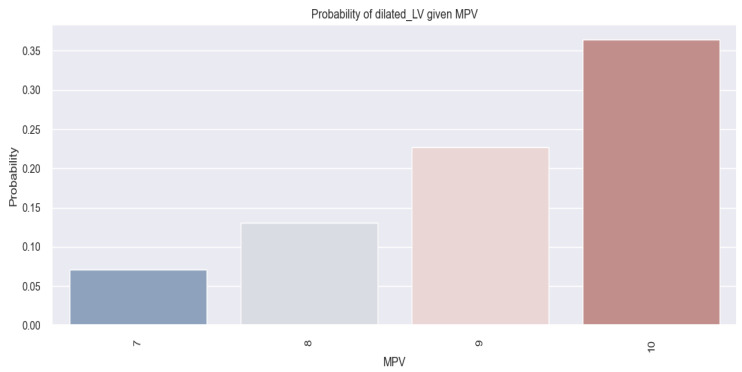
Estimated probability of HF patients having a dilated LV based on their MPV value, using the logistic regression method.

At the same time, the identification of a relationship between a higher MPV value and a dilated LV is exemplified clearly in the figures below (Figure 17 and Figure 18). From the graphic representations in Figure 17, we can observe that patients with a dilated LV had higher MPV values (over 9 fl) in comparison to those without a dilated LV.

These results are better delineated through the application of the logistic regression model, whose data are graphically represented in Figure 18. Through this model, we can estimate the possibility of an HF patient having a dilated LV based on their MPV value. So, the figure below (Figure 18) reveals that there is an increased tendency for a patient with HF to have a dilated LV as their MPV value increases (for example, in patients with an MPV of 10 fl, there is a 35% chance of them having a dilated LV, in comparison to patients with an MPV value of 7 fl, for whom this chance is below 10%).

In summary, considering the arguments presented above, it can be affirmed that the MPV value serves as a predictor for the probability of an HF patient having a dilated LV. Furthermore, our study reveals that the higher the MPV value, the greater the probability of a patient with HF having a dilated LV.

#### 3.3.4. Relationship between MPV and PH

**Figure 19:**
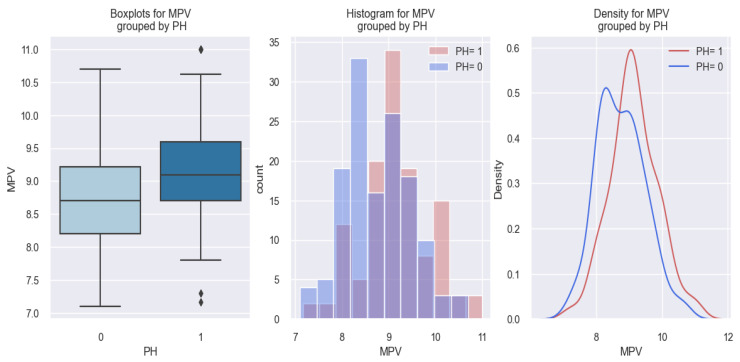
MPV values in HF patients with PH (PH = 1) versus HF patients without PH (PH = 0).

**Figure 20:**
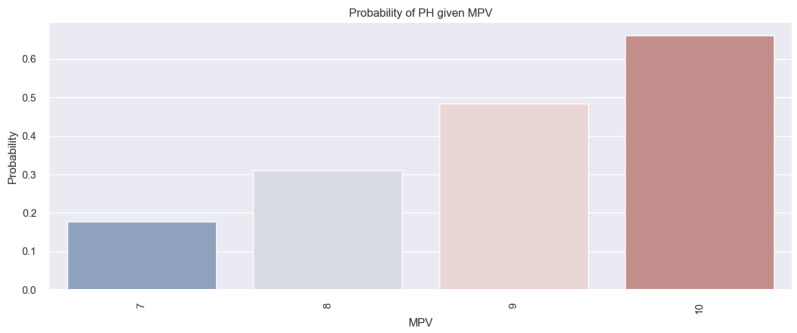
Estimated probability of having PH depending on MPV value in the studied population.

Our study revealed that patients with pulmonary hypertension (PH) had a higher value of MPV compared to those without PH. This phenomenon is exemplified in the figures below (Figure 19 and Figure 20).

In the first graph of Figure 19, it can be observed that the presence of PH (PH = 1) is associated with an increased MPV value in comparison to the absence of PH (PH = 0) in our studied population, and from the following two graphs, it can be seen that patients with PH are distributed more towards the right, so towards higher MPV values, hence there is a slight tendency for MPV values to be higher for patients with PH.

The insights derived from the above results are better described through the logistic regression model, whose data are represented in Figure 20. This model provides an estimation of the probability of an HF patient having PH based on their MPV value. Figure 20 highlights an increased tendency for PH to be present in HF patients with high MPV values. For instance, there is an over 60% chance that PH will be present in patients with an MPV of 10 fl in comparison to patients with an MPV of 8 fl (where there is a 31% chance of PH presence). In conclusion, based on the results presented above, it can be asserted that the MPV value can be used to predict the probability of an HF patient having PH. Moreover, in Figure 20, it can be observed that the higher the MVP value, the greater the probability of an HF patient having PH.

## 4. Discussion

Through our research, we aimed to add novelty to the current medical research regarding MPV as a prognostic parameter in HF patients by identifying correlations between MPV values and certain parameters, such as increased NT-proBNP, increased RDW, and the presence of an AFib rhythm, reduced LVEF, dilated LA and LV, and PH, which are considered negative prognostic variables in HF patients. 

Based on the above correlations, MPV could be used as a prognostic parameter in populations of patients with HF, and through it, we could gain the ability to identify patients with HF at risk of having a negative prognosis. 

Regarding the identification of a statistical link between the value of NT-proBNP and the value of MPV, our study confirms the results of the research conducted by Budak and his collaborators [17], which focused on patients with HF, and which revealed that BNP values were positively correlated with MPV values. The results of our research showed that HF patients with higher NT-proBNP values had higher MPV values, with the differences between our research and the study conducted by Budak being as follows: (1) our study focused on the relationship between NT-proBNP and MPV values; (2) the relationship between MPV and NT-proBNP values was demonstrated by applying the linear regression model, which revealed that by increasing the MPV value by one unit, NT-proBNP is increased by 2677 pg/mL.

A possible explanation for the correlation between increased levels of MPV and increased levels of NT-proBNP is that since NT-proBNP is a biomarker released as a consequence of the activation of compensatory neurohormonal mechanisms in decompensated heart failure, and thus is a biomarker linked to the aggravation of the clinical status in HF patients, this is the reason why HF patients with increased levels of NT-proBNP may also have increased levels of MPV.

Regarding the MPV–presepsin relationship in patients with HF, the studies are lacking, and the only ones available in the current medical literature are those that refer to the correlation between patients with sepsis and increased values of MPV, as shown by the study conducted by J. Van Der Lelie [18], who, however, did not target a population of patients with HF and did not follow the interrelation between presepsin and MPV. The explanation according to which patients with sepsis would have an increased MPV value is that the infection is predisposed to induce the appearance of immune complexes and the release of cytokines, which determines, through a compensatory mechanism, the occurrence of thrombocytopenia, expressed biologically by increased levels of MPV [9]. However, regarding our statistical results regarding the relationship between presepsin and MPV values, our study did not find a statistically significant relationship between these variables, the most plausible cause of this phenomenon being the reduced number of patients for which presepsin data were available.

Regarding the relationship between MPV and LVEF, two studies are cited in the literature: the one conducted by Nassiba M. et al. [19] and the one conducted by Schu-ichi Fujita et al. [20]. The study conducted by Nassiba M. [19] revealed that patients with a preserved LVEF had a higher MPV and that a larger MPV is considered a negative prognostic marker in HF patients with a preserved LVEF. The novelties brought by our research in contrast to the study conducted by Nassiba M. are as follows: (1) our research was focused on a population of HF patients regardless of their LVEF value; (2) our statistical results outlined that HF patients with a reduced LVEF had increased MPV values in comparison to those with a preserved LVEF, who had low values of MPV. The study conducted by Schu-ichi Fujita et al. [20] concentrated on a population of patients with both systolic and diastolic LV dysfunction, revealing that MPV is an independent factor associated with both systolic and diastolic LV dysfunction. The differences brought by our study are that patients with LV diastolic dysfunction (preserved LVEF) had a lower MPV value compared to patients with LV systolic dysfunction (reduced LVEF), who had higher MPV values, and that our results were obtained based on the application of logistic regression models, through which it was demonstrated that as their MPV value increases, an HF patient will be more likely to have a reduced LVEF. The possible reason for which MPV levels may be higher in patients with a reduced LVEF is that a lower LVEF is correlated to increased filling pressures in the left ventricle; this activates the neurohormonal compensatory mechanism, which is linked to a greater status of decompensation in heart failure, and as a consequence, the levels of MPV increase. 

Regarding the relationship between MPV and AFib rhythms, the results of our research confirm other data from the medical literature according to the studies conducted by Yucel Colkesen [21] and by Okan Turgut et al. [13], according to which patients with a higher MPV value are at higher risk of paroxysmal AFib, and patients with an AFib rhythm had a higher MPV compared to patients with SR, respectively. The novelty of our study is the study population (patients with HF), as well as the statistical methodology applied (logistic regression models), through which we demonstrated that the higher the MPV value, the higher the probability of a patient with HF having AFib. The possible mechanism behind this link between the presence of AFib and increased levels of MPV in heart failure patients is because the AFib rhythm is one of the atrial arrhythmias associated with increased filling pressures in the left atrium and left ventricle, which in turn increase the pressure in pulmonary veins and capillaries, which consequently will be responsible for pulmonary congestions. The above chain of reactions will be responsible for a greater risk of decompensation in heart failure and for the activation of the neurohormonal and inflammatory compensatory systems, which in turn will produce platelet dysfunction, expressed by increased levels of MPV.

Extrapolating our statistical results according to which patients with a dilated LA had a higher value of MPV compared to patients with a non-dilated LA, we can link them to the statistical results obtained in the case of the relationship between the presence of an AFib rhythm and the value of MPV, taking into account that it is well known in the medical literature that patients with an AFib rhythm have a dilated LA due to the remodeling process through which their LA undergoes.

At the same time, by extrapolating the statistical results according to which patients with a dilated LV had a higher value of MPV compared to patients with a non-dilated LV, these interesting data can be linked to the statistical results obtained in the case of the relationship between LVEF and the value of MPV, taking into account that it is known in the medical literature that the majority of patients with a dilated LV have a reduced LVEF.

Regarding the results from our study according to which patients with HF with increased MPV values had a higher probability of having higher RDW values, this represents a novelty in the medical literature since the available studies related to this aspect are limited, and the existing studies on HF and MPV or HF and RDW did not study the possible statistical correlation between MPV and RDW values in patients with HF. 

The current available medical research regarding the impact of RDW on HF patients is cited in two studies: one of them is a review-type study conducted by Andrew Xanthopoulos et al. [22], who revealed that patients with a higher RDW were more likely to have decompensated HF, and the second one is a type of a meta-analysis study conducted by Yuan-Lan Huang et al. [23], which revealed that HF patients with high RDW values had a more reserved prognosis, represented by frequent hospitalizations or increased mortality. The novelty that our study adds to the medical field in this context is that our research focused on MPV value–RDW value correlation in the HF population, revealing that patients with increased MPV values had increased RDW values. Therefore, the results from our study regarding the identification of a linear relationship between MPV and RDW values are promising. The reason why our findings revealed a link between elevated RDW values and elevated MPV values is probably because, like MPV, RDW also increases as a result of activation of the neurohormonal and inflammatory compensatory systems in heart failure patients, which in turn is responsible for anisocytosis, the latter one being responsible for increased levels of RDW [22,23].

The main strengths of our research are provided by the statistical analysis used, which was represented by regression model analyses. Through the above statistical analyses, we could determine significant correlations between MPV values and certain biological, electrocardiographic, and echocardiographic variables that are considered negative prognostic parameters in HF patients. Based on these promising results, we could consider that the MPV value may be a negative prognostic parameter in HF patients, and based on its value, we could target HF patients at risk of a poor outcome. 

Since increased NT-proBNP, reduced LVEF, increased RDW, AFib, PH, and dilated LV and LA are variables associated with a worse clinical status and outcome in heart failure patients according to the available medical literature, and since our results revealed correlations between increased MPV values and these variables, we could expect that an increased MPV may predict negative changes in these biological, electrocardiographic, and echocardiographic parameters, and thus predict a negative prognosis in heart failure patients. 

MPV is a parameter that is easy to obtain and because it is incorporated in any hemogram, it is available for any practitioner. The fact that a single parameter such as MPV could predict changes in the variables mentioned above and hence enable estimations of the probability of poor prognoses in heart failure patients represents an element of novelty for clinical practices, because through it we can label a patient at risk of a poor outcome and start a premature aggressive treatment to prevent future decompensations.

There are several limitations associated with our study:

(1) Retrospective design: this study was conducted retrospectively, involving data collection from the system database of the hospital; this approach introduces the potential of biased patient selection, as retrospective studies rely on pre-existing data, and the selection of patients may not be randomized.
(2) Influence of blood sample collection: NT-proBNP, RDW, and mean platelet volume values may be impacted by the retrospective method of blood sample collection; human error during these procedures may introduce variations in the recorded values, potentially affecting the accuracy and reliability of the results.

## 5. Conclusions

Based on the results from our study, we can conclude that there is a statistically significant relationship between increased values of MPV (over 9 fl) and biological (increased NT-proBNP, increased RDW), electrocardiographic (presence of AFib rhythm), and echocardiographic variables (reduced LVEF, dilated LV, dilated LA, presence of PH) in the studied population of patients with HF. Therefore, we could consider that MPV may be used as a negative prognostic parameter in HF patients, and based on its value, we may be able to identify heart failure patients susceptible to a negative prognosis.

## Figures and Tables

**Table 1:** Normal values of biological and echocardiographic variables.

Variable	Normal Value
NT-proBNP	<125 pg/mL
Presepsin	<300 mg/dL
RDW	<14%
MPV	7–8 fl
Reduced LVEF	<50%
Preserved LVEF	>50%
LA diameter	22–41 mm
LV diameter	37–50 mm

**Table 2:** The relation between the MPV value and NT-proBNP value in the studied population.

Biomarker:	NT-proBNP	R-Squared:	0.091			
	Coefficient	Standard Error	t	*p* Value > |t|	[0.025	0.975]
Intercept	−17,610	4730.928	−3.721	0	[−26,900	−8288.88]
MPV	**2677.3154**	527.613	5.074	**0**	[1638.339	3716.291]

**Table 3:** The relation between MPV and presepsin values in the studied population.

Biomarker:	Presepsin					
No. of Observations:	39					
	Coefficient	Standard Error	t	*p*-Value > |t|	[0.025	0.975]
Intercept	2272.6483	835.742	2.719	0.01	[579.275	3966.022]
MPV	**−182.7411**	90.047	−2.029	**0.05**	[−365.195	−0.288]

**Table 4:** MPV–RDW value relationship, determined with the linear regression model, in HF patients.

Biomarker:	RDW	R-Squared:	0.037			
	Coefficient	Standard Error	t	*p*-Value > |t|	[0.025	0.975]
Intercept	9.5573	1.244	7.685	0	[7.108	12.006]
MPV	**0.4377**	0.139	3.156	**0.002**	[0.165	0.711]

**Table 5:** The relation between MPV value and the probability of AFib, determined with the logistic regression model, in HF patients.

Variable:	AFib	No. of Observations:	260			
	Coefficient	Standard Error	Z	*p*-Value > |z|	[0.025	0.975]
Intercept	−4.8282	1.573	−3.069	0.002	[−7.912	−1.745]
MPV	**0.5261**	0.175	3.002	**0.003**	[0.183	0.87]

**Table 6:** The relation between MPV value and the likelihood of SR, determined with the logistic regression model, in HF patients.

Variable:	SR	No. of Observations:	260			
	Coefficient	Standard Error	Z	*p*-Value > |z|	[0.025	0.975]
Intercept	4.9591	1.578	3.143	0.002	[1.866	8.052]
MPV	**−0.5372**	0.176	−3.057	**0.002**	[−0.882	−0.193]

**Table 7:** The relation between MPV and LVEF, determined with the linear regression model, in HF patients.

Variable:	LVEF	R-Squared:	0.053			
	Coefficient	Standard Error	T	*p*-Value > |t|	[0.025	0.975]
Intercept	80.9093	9.228	8.768	0	[62.738	99.08]
MPV	**−3.9016**	1.029	−3.791	**0**	[−5.928	−1.875]

**Table 8:** The relation between MPV value and reduced LVEF, determined with the logistic regression model, in HF patients.

Variable:	Reduced LVEF	No. of Observations:	260			
	Coefficient	Standard Error	Z	*p*-Value > |z|	[0.025	0.975]
Intercept	−5.1414	1.587	−3.24	0.001	[−8.251	−2.032]
MPV	**0.5485**	0.176	3.109	**0.002**	[0.203	0.894]

**Table 9:** The relation between MPV value and preserved LVEF, determined with the logistic regression model, in HF patients.

Variable:	Preserved LVEF	No. of Observations:	260			
	Coefficient	Standard Error	Z	*p*-Value > |z|	[0.025	0.975]
Intercept	5.4001	1.595	3.386	0.001	[2.275	8.526]
MPV	**−0.581**	0.177	−3.275	**0.001**	[−0.929	−0.233]

**Table 10:** The relation between MPV value and LA diameter, determined with the linear regression model, in HF patients.

Variable:	LA Diameter (mm)	R-Squared:	0.069			
No. of Observations:	260	AIC:	1700			
	Coefficient	Standard Error	T	*p*-Value > |t|	[0.025	0.975]
Intercept	27.5822	4.727	5.835	0	[18.273	36.891]
MPV	**2.3035**	0.527	4.369	**0**	[1.265	3.342]

**Table 11:** The relation between MPV value and LV diameter, determined with the linear regression model, in HF patients.

Variable:	LV Diameter (mm)	R-Squared:	0.048			
No. of Observations:	260	AIC:	1622			
	Coefficient	Standard Error	T	*p*-Value > |t|	[0.025	0.975]
Intercept	36.1691	4.067	8.893	0	[28.16	44.178]
MPV	**1.6395**	0.454	3.614	**0**	[0.746	2.533]

## Data Availability

The data presented in this study are available on request from the corresponding author. The data are not publicly available due to ethical restrictions.

## Appendix A. Types of Variables Used

**Independent Variable**	**Dependent Variable**	**Type of Variable**	**Regression Type**
MPV	NT-proBNP	Continuous	Linear
MPV	Presepsin	Continuous	Linear
MPV	RDW	Continuous	Linear
MPV	AFib	Binary	Logistic
MPV	SR	Binary	Logistic
MPV	LVEF	Continuous	Linear
MPV	Reduced LVEF	Binary	Logistic
MPV	Preserved LVEF	Binary	Logistic
MPV	LA diameter_(mm)	Continuous	Linear
MPV	LV diameter (mm)	Continuous	Linear
MPV	Dilated LV	Binary	Logistic
MPV	Dilated LA	Binary	Logistic
MPV	PH	Binary	Logistic
